# Hahnemnan the Founder of Isopathy

**Published:** 1883-12

**Authors:** S. Swan


### HAHNEMANN, THE FOUNDER OF ISOPATHY!

Mr. Editor:—Your anti-nosode contributor will please read *Chronic Diseases*, Vol. I, page 196, and he will see that he is wrong in attributing to me the honor of discovering that which Hahnemann stated so clearly in 1828, to this effect: that preparation, *i. e.*, trituration, dilution, succussion, potentization of nosodes, or morbific products, *so altered their nature to that of a homœopathic remedy that without such alteration they could not have any effect upon an organism tainted with the same identical virus*, which is equivalent to saying that *if* so altered they *would* have an effect. This is all I claim—that these potentized morbific products *become homœopathic to the poison of the same virus in a human organism.* I cannot understand Hahnemann's statement otherwise.

S. Swan.

